# Return to work after volar or combined plating of type C distal radius fracture: a secondary analysis of a randomised clinical trial

**DOI:** 10.1186/s12891-025-09447-5

**Published:** 2025-12-30

**Authors:** Erik Noppa, Marcus Sagerfors, Eva Lundqvist

**Affiliations:** 1 Department of Anaesthesiology and Intensive Care, Örebro University Hospital, Södra Grev Rosengatan, Örebro, Region Örebro County SE-70185 Sweden; 2 Department of Orthopedic and Hand Surgery, Örebro University Hospital, Örebro, Region Örebro County Sweden; 3 School of Medical Sciences, Faculty of Medicine and Health, Örebro University, Örebro, Sweden

**Keywords:** Distal radius fracture, Patient-related outcome measures, Plating, Wrist function, Sick leave, Rehabilitation, Health economics

## Abstract

**Background:**

Distal radius fracture (DRF) is the most common of fractures, accounting for 18% of all fractures. The ability to return to work is an important outcome for both society and the individual, but there is a large variation among patients in how much time off work is required. The choice of treatment is likely an important factor.

**Methods:**

This study was a secondary analysis of an earlier published randomised clinical trial comparing volar locking plate fixation with combined dorsal and volar plate fixation of AO type C DRF. In the present study, we examined the the duration of sick leave among patients who were in the workforce at the time of fracture.

**Results:**

There were 84 cases included in this analysis.Combined plating was associated with a significantly longer time to return to work following a type C DRF, in comparison to treatment with a volar locking plate. The crude difference was 0.67 months (*p* = 0.33; 95% CI: 0.85–1,26 months), and 0.65 months (*p* = 0.15; 95% CI: 0.13–1.17) when adjusting for differences in manual labour indicating a statistically significant difference time until return to work.

**Conclusions:**

Sick leave is an important contributor to total costs in patients with DRF, and more invasive treatments like combined plating are associated with longer sick leave. Our results indicate a longer time until return to work in the combined plating group, possibly due to more extensive soft tissue dissection. We recommend a restrictive use of combined plating.

**Trial registration:**

The study was registered in the Swedish research database FoU in Sweden (registration number: 274674) on the 4th of August 2020.

## Background

The most common fracture in the adult population is the distal radius fracture (DRF), which has an incidence of 278 fractures per 100,000 person-years and accounts for 18% of all fractures [1, 2]. Orthopaedic trauma is associated with socioeconomic impact for the patient and society, and the mean length of absence from work following a DRF is about 90 days [3]. However, there is significant variability in patients’ needs, with 21–31% taking no time off work at all [4, 5]. The most important factors associated with a long absence from work include high levels of pain, poor self-assessed disability 1 week after treatment, need for surgical treatment, and high occupational demands [4, 5]. High age and an initially displaced fracture are also risk factors [4]. Educational level does not affect the length of absence from work when corrected for occupational demands [4, 5]. All upper extremity fractures, including DRF, require more time absent from work than finger fractures; but one study found that for all upper extremity fractures, socioeconomic factors such as lack of fixed employer and feeling economic distress were associated with unsuccessful return to work within 90 days [6]. Studies comparing the impact of surgical technique on time to return to work has shown no, or a slight advantage of volar locking plate compared to intramedullar nailing or pinning [7–9]. To our knowledge, no studies have examined time to return to work following volar locking plate fixation compared to combined dorsal and volar plate fixation for patients with AO type C DRF.

During the normal course following DRF, severe symptoms recede in 2 months, and only minimal pain and disability are present at 6 months [10]. Although the majority of patients recover with good function, 17% still have major disabilities 1 year after a fracture [11]. Complications following surgical treatment may delay this recovery significantly, and interference with tendons, tendon irritation, and the need for hardware removal are commonly described problems with volar and combined plating [12, 13]. Today, about 50% of Arbeitsgemeinschaft für Osteosynthesefragen (AO) type C DRFs are treated surgically, most commonly with volar plating [14–16]. A meta-analysis of trials comparing non-operative treatment to volar locking plate, in which the majority of fractures were AO type C DRF, demonstrated that volar plating in these patients led to improved function at 3 months, 6 months, and 12–24 months after fracture [17]. However, in some complex cases of the most comminuted AO type C DRF, a volar plate alone may not provide sufficient stability [18]. 

We have previously published a randomised clinical trial (RCT) comparing volar locking plate fixation with combined dorsal and volar plate fixation of AO type C DRF. We found significantly better patient-reported outcomes, grip strength, and range of motion (ROM) in the volar plate locking plate group, but the between-group difference in Quick Disabilities of the Arm, Shoulder, and Hand (QuickDASH) score was smaller than the minimal clinically important difference. No difference in radiological outcomes was found [19]. 

Since combined plating is associated with more extensive soft tissue dissection, longer operating time, and an increased risk of infection [20, 21], we hypothesised that it could also be associated with a longer time until return to work. This study aimed to investigate whether there is a difference in time to return to work with combined plating compared to volar plating.

## Methods

This was a secondary analysis of a previously published RCT. The study sample consisted of adult patients (≥18 years) with AO type C fractures treated at Örebro University Hospital, a tertiary referral hospital, between June 2017 and July 2019. Inclusion and exclusion criteria are listed in Table 1. The study was approved by the regional ethical committee (ref: 2016 − 455), and the study was registered in the Swedish research database, FoU in Sweden (registration number: 274674). All patients gave written informed consent before participation according to the Helsinki Declaration [22]. 

**Table 1:** Inclusion and exclusion criteria

Inclusion criteria	Exclusion criteria
Age 18–80 years	Previous fracture of the same wrist
Operation within 12 days of injury	Bilateral fractures
AO type C with one or more of the following:	Other concomitant fractures
> 20° dorsal angulation of the distal radial articular surface	Open fracture
	Fracture extending to the diaphysis
> 2 mm ulnar plus	
	Ongoing chemotherapy or radiotherapy
	Metabolic diseases that affect bone
	Dementia
	Mental illness
	Alcohol misuse disorder or opiate addiction
	Difficulty understanding Swedish
	Severe neurological disease
	Severe cardiopulmonary disease
	Not in workforce at time of injury

*AO* Arbeitsgemeinschaft für Osteosynthesefragen

A total of 150 patients (*n* = 75 in each group) were randomized to open reduction and fixation using a volar locking plate (TriMed) or combined plating with a volar T-plate and a low-profile dorsal plate with variable angle locking screws (TriMed). All surgeries were performed by specialists in hand surgery. The patients were randomized using sealed opaque envelopes containing the study number and surgical technique, which were opened prior to surgery by the operation room nurse.

The sample size for the original study was calculated on the basis of the QuickDASH score [19]. A post-hoc power analysis for this secondary analysis demonstrated a 62% power for the detection of a 0.5 month increase in time to return to work, assuming a standard deviation of 1 month.

### Surgical technique

All surgeries were performed under combined brachial plexus block and general anaesthesia. A tourniquet was used. A volar central incision was made to expose the volar ulnar portion of the distal radius, and the carpal tunnel was opened [23]. This surgical approach was clinical routine for AO type C DRFs at the time of the study. Instructions on active finger ROM exercises and oedema control by a hand therapist on the first postoperative day [19]. 

**Fig. 1:**
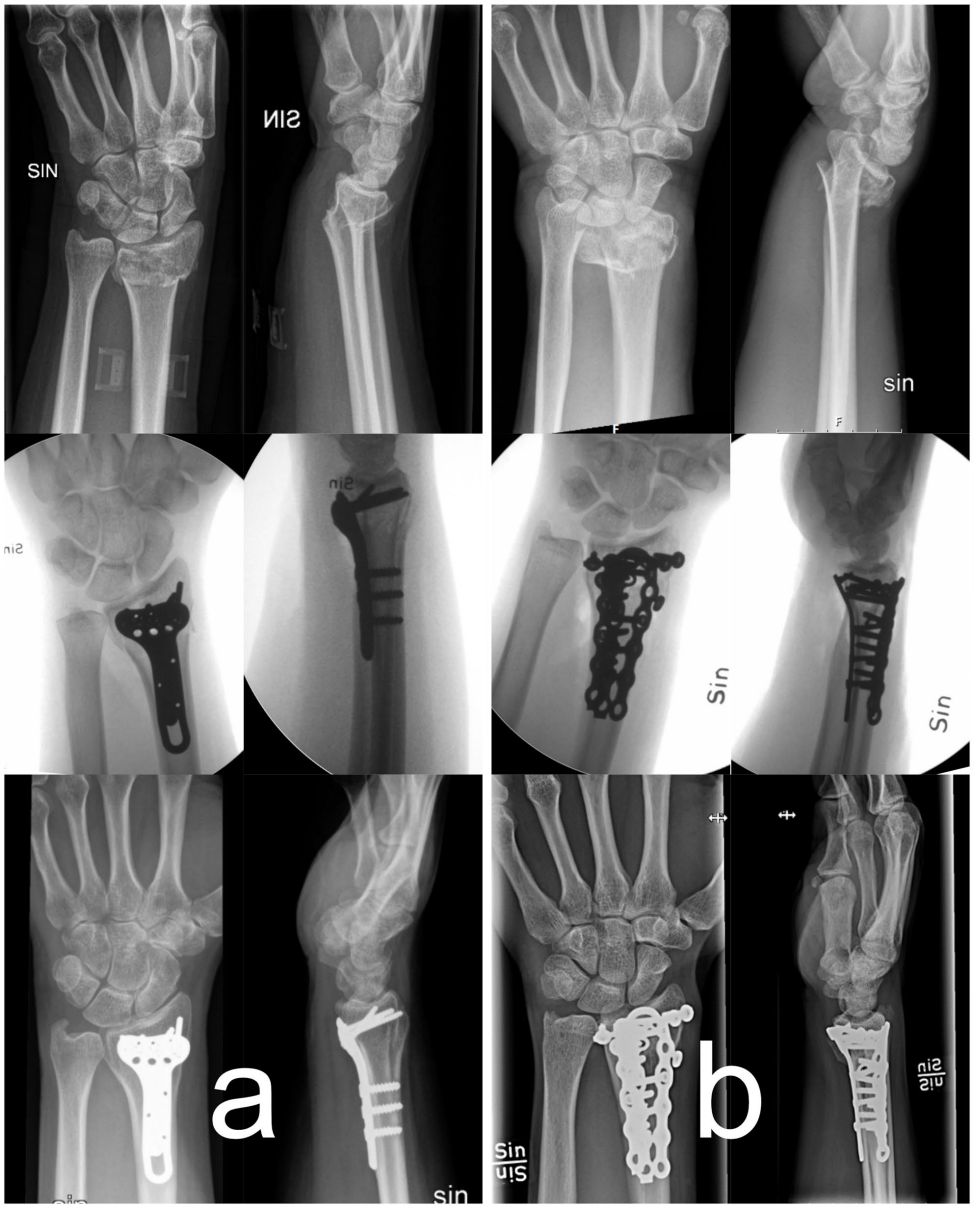
Radiographs preoperative, interoperative, and at 12-moths follow-up. **a** volar plating, **b** combined plating

In the combined plating group, an additional dorsal longitudinal incision was made centrally over Lister’s tubercle (Fig. 1).

If an associated ulnar styloid fracture was present, the stability of the distal radioulnar joint (DRUJ) was assessed after plate fixation of the DRF. DRUJ instability was treated with reduction and fixation of the styloid with either a 3.0-mm cannulated screw (DePuy Synthes) or a 2.0-mm locking ulna hook plate (DePuy Synthes). Regardless of surgical method a cast was applied for 2 weeks, followed by an orthosis for 2 weeks. Mobilization was gently initiated 2 weeks after the surgery.

Antibiotic prophylaxis (intravenous cloxacillin 2 g, or in case of intolerance to β-lactam antibiotics, clindamycin 600 mg) was used if the duration of surgery exceeded 2 h or if wounds were present in or near the surgical field.

### Clinical evaluation

At the 1-year follow-up, a hand therapist performed clinical measurements and patient-reported outcome measurements [19]. Pain was evaluated both at rest and during activity using the VAS pain score (0 = no pain, 10 = worst imaginable pain).

Data on the duration of time of work and manual labour were collected from patient data records and patient interviews at the 12-month follow-up. Having spent more than 3 months off work was considered as delayed return to work. The type of work was defined as manual or non-manual, based on the Swedish Standard Classification of Occupations (SSYK 2012), which in turn is based on the International Standard Classification of Occupations (ISCO-08) [24, 25]. 

### Statistical analysis

Statistical analyses were performed using version 29.0 of IBM SPSS Statistics for Windows. Demographic data are presented as the number of cases with median and interquartile range (IQR). The Shapiro-Wilk test was used to assess normality of distribution (data not shown). Because the data were not normally distributed and ordinal data were used, the results are presented as median and IQR when this is most appropriate. Comparisons between groups were made using the Mann-Whitney test, Student’s t-test, χ^2^ test, and Fisher’s exact test as appropriate.

We also performed a multivariable linear regression to estimate the effect of surgical method while adjusting for manual labour. P-values < 0.05 were considered statistically significant.

## Results

**Fig. 2:**
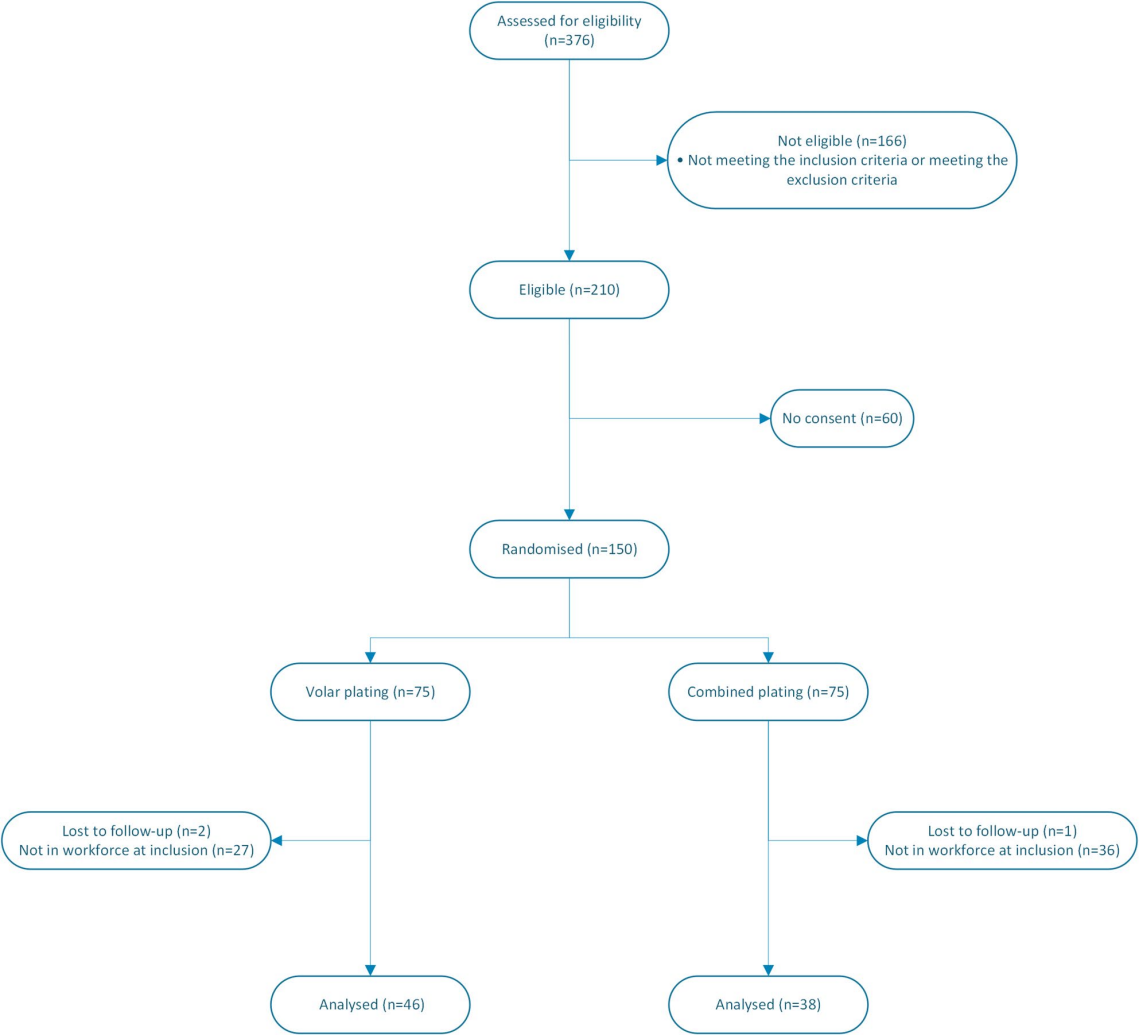
CONSORT flow-chart

Of the 84 cases available for this secondary analysis (Fig. 2), there were no statistically significant differences in baseline data between the treatment groups (Table 2). The average time of work in the volar plating group was 2.4 (median 3.0; IQR 2.6–3.0; range 0.0–4.0), months compared to 3.1 (median 3.0; IQR 3.0–3.0; range 0.0–10.0) months in the combined plating group (*p* = 0.33).

**Table 2:** Baseline characteristics of the treatment groups

	Volar plating	Combined plating	Total	*p*-value
Age	53 (47–61)	54 (44–59)	53 (45–60)	NS
Sex, M/F	10/36	10/28	20/64	NS
Side, dx/sin	21/25	18/20	39/45	NS
Fracture subtype, C1/C2/C3	12/19/15	10/17/11	22/36/26	NS
Trauma type, low/high energy	34/12	29/9	63/21	NS
Injury of dominant hand, yes/no	17/29	17/22	33/51	NS
Manual work, yes/no	30/16	25/13	55/29	NS
Operating time in minutes	59 (42–74)	85 (71–96)	70 (51–88)	< 0.001

A χ^2^ test was used for comparison, except for age and operating time, where the Mann-Whitney U-test was used. Age, and operating times is given as median and IQR

We also performed a multivariable linear regression, including operation method and manual labour (Table 3). When added to the multivariable model, sex, age, trauma type, or pain at rest or during activity were not statistically significant. No statistically significant interaction was present.

**Table 3:** Multivariable linear regression

Variable (univariate)	Coefficient (β)	SE	*p*-value	95% CI
Surgical method	**0.65**	**0.26**	**0.015**	**0.13–1.17**
Manual labour	**1.48**	**0.27**	**< 0.001**	**0.94–2.03**

*CI* Confidence interval

In a sensitivity analysis, the data was analysed regarding the risk of return to work being > 3 months using a logistic regression model. This risk was significantly higher in the combined plating group, with 8 patients failing to return to work within 3 months compared with 2 patients in the volar plate group.

## Discussion

In this secondary analysis, we found that combined plating was associated with a significantly longer time to return to work following a type C DRF, in comparison to treatment with a volar locking plate. The crude difference was 0.67 months (*p* = 0.33; 95% CI: 0.85–1,26 months), and 0.65 months (*p* = 0.15; 95% CI: 0.13–1.17) when adjusting for differences in manual labour indicating a statistically significant difference time until return to work.

Sick leave is an important contributor to total costs in patients with DRF, and earlier retrospective studies have shown that more invasive treatments are associated with longer sick leave [4, 26]. This is well in line with our results. As the clinical outcomes in the previously published RCT indicated similar radiographic outcomes and inferior clinical outcome parameters for combined plating. The findings in this present analysis indicate no clinical advantage from routinely use of combined plating over volar plating.

The original RCT comparing the use of a volar locking plate with the use of combined dorsal and volar plates for surgical treatment of AO type C DRFs demonstrated significantly inferior QuickDASH and PRWE scores in the combined plating group [19]. Neither of these patient-reported outcomes was significantly associated with time to return to work in this analysis. However, an inferior outcome regarding delayed return to work for the combined plating group is in line with earlier published results.

Pain is an important factor for outcome after hand surgery, but pain is also linked to anxiety, personality, and catastrophic thinking and not only a function of trauma and the surgical procedure [27, 28]. In this study, pain levels was not associated with time to return to work.

In the present study, we analyse the data as a continuous variable. However, the Swedish medical insurance guidelines recommend 5–12 weeks of sick leave, depending on the need for rehabilitation and physical demands at work [29]. This means that the majority of surgically treated type C DRF patients return to work at around 3 months, most likely due to the social benefits system rather than fracture healing. This is clear in our data with despite the differences in time to return to work the median is 3.0 months in both treatment groups, which might lead to underestimation of the difference in physical ability. In Sweden, the Swedish Social Insurance Agency grants sick-leave based on the recommendation of the patient’s physician. However, the decision to return to work earlier can be made collaboratively by the worker and employer, even if the approved sick-leave period has not ended. Other systems of deciding when to return to work as well as other levels of compensation from the health insurance system is likely to yield somewhat other results. The results of this study may therefore be most relevant in countries with similar social benefits systems; however, the risk of prolonged recovery is likely higher in combined plating regardless of the social benefits system.

Except for the comparison between two different surgical methods, no other interventions were implemented to shorten the time off work. In one study of metacarpal and phalangeal fractures, interventions at the workplace shortened the time off work from 56.3 days to 4.8 [30]. The effect of these kinds of interventions, regardless of surgical treatment, is an area for further research in DRF.

A limitation in this study is the lack of socioeconomic factors other than work. This might have introduced bias, but the RCT design should have limited the effect. The impact of socioeconomic factors could be a topic for future studies, preferably using register data. Another limitation reducing the generalizability of the findings, is that all patients were operated using a volar central incision including a carpal tunnel release, instead of the more commonly used Henry incision.

A thorough cost analysis would have been interesting to perform but the original study lacks data on personnel use/costs and the duration of in-hospital care. However, all available results impacting such an analysis are neutral or in favour of volar plating. Shorter surgical time, no use of an additional plate, lower infection and hardware removal frequencies, and shorter sick leave all indicate this.

Further research in this area could be focused on the impact of other surgical or non-surgical treatment options for DRF. Another area to study would be the effect of hand therapy, or of different interventions in the workplace.

## Conclusions

The results of this secondary analysis indicate that combined plating is associated with longer time until return to work following a type C DRF when compared to volar plating alone. A restrictive use of combined plating and perhaps of other more invasive surgical techniques is recommended based on these findings.

## Data Availability

The datasets used and analysed during the current study are available from the corresponding author upon reasonable request.
